# Heuriskance: a novel paradigm for systematic earlier detection of sporadic pancreatic cancer

**DOI:** 10.1093/jnci/djaf291

**Published:** 2025-10-08

**Authors:** Suresh T Chari, Ziding Feng, Bechien Wu, William Fisher, Avinash Kambadakone, Ying-Qi Zhao, Anirban Maitra, Barbara Kenner, Lynn M Matrisian

**Affiliations:** Department of Gastroenterology, University of Texas MD Anderson Cancer Center, Houston, TX, United States; Public Health Sciences Division, Fred Hutchinson Cancer Center, Seattle, WA, United States; Department of Gastroenterology, Kaiser Permanente Southern California, Los Angeles, CA, United States; Department of Surgery, Baylor College of Medicine, Houston, TX, United States; Department of Radiology, Massachusetts General Hospital, Boston, MA, United States; Public Health Sciences Division, Fred Hutchinson Cancer Center, Seattle, WA, United States; Sheikh Ahmed Center for Pancreatic Cancer Research, University of Texas MD Anderson Cancer Center, Houston, TX, United States; Kenner Family Research Fund, New York, NY, United States; Pancreatic Cancer Action Network, El Segundo, CA, United States

## Abstract

Early detection is key to improving survival and mortality from pancreatic cancer. Traditional periodic screening for cancer in an asymptomatic population is infeasible and not recommended for this low-incidence disease. We describe a novel approach we call “heuriskance” (*hyou-ris-kance*), wherein a systematic search for and 1-time workup of a “heurisk” *(hyou-risk)* leads to earlier detection of cancer. A heurisk is an early-warning sign with 3 defining characteristics: (1) the individual has a higher-than-threshold probability of having prevalent invasive cancer, (2) it is associated with a meaningful lead time to diagnosis, and (3) it is identifiable by a systematic and scalable process in the population. Heuriskance aims to systematically detect cancer with clinically meaningful lead time to clinical diagnosis, minimize the proportion of patients with advanced disease, and maximize treatment options, leading to increases in lead time–adjusted 1-, 3-, and 5- year survival. A specific example of a heurisk for pancreatic cancer is glycemically defined new-onset diabetes and the Early Detection Initiative for Pancreatic Cancer (ClinicalTrials.gov identifier NCT04662879) an example of glycemically defined new-onset diabetes-based heuriskance. As heuriskance has no precedent, we provide (1) a tiered risk stratification approach (Define-Enrich-Find), (2) metrics for choosing a heurisk, (3) success metrics for strategy, and (4) phases 1-5 for evaluating the strategy in retrospective and prospective studies. Like all current cancer therapies, heuriskance aims to iteratively improve survival from a fatal disease using a pragmatic, evidence-based, systematic approach to its earlier detection. We apply the concept of heuriskance to pancreatic cancer, but it could be extended to other cancer types.

## Introduction

Pancreatic cancer is the 10th-most common cancer in the United States but by 2030 is projected to become the second-most common cause of cancer death.^1^^,^^2^ At diagnosis, approximately 50% of pancreatic cancers are stage IV, and approximately 85% are unresectable. If the disease is localized, 5-year survival is 40% compared with less than 3% in individuals with metastatic disease.^2^ Although surgical resection of early cancer provides the greatest hope for cure, chemotherapy is the mainstay of treatment for all patients with pancreatic cancer who can tolerate it. The extension of life through chemotherapy is valued by patients and loved ones, even if pancreatic cancer remains the cause of death. Key limiting factors to offering aggressive chemotherapy and improving survival are advanced-stage disease, extensive tumor burden, and poor performance status, all of which can be favorably affected by earlier detection. Earlier detection of resectable disease in individuals without noticeable symptoms is the cornerstone of improving survival, and eventually mortality, in pancreatic cancer.

## Screening for pancreatic cancer

**Figure 1.**
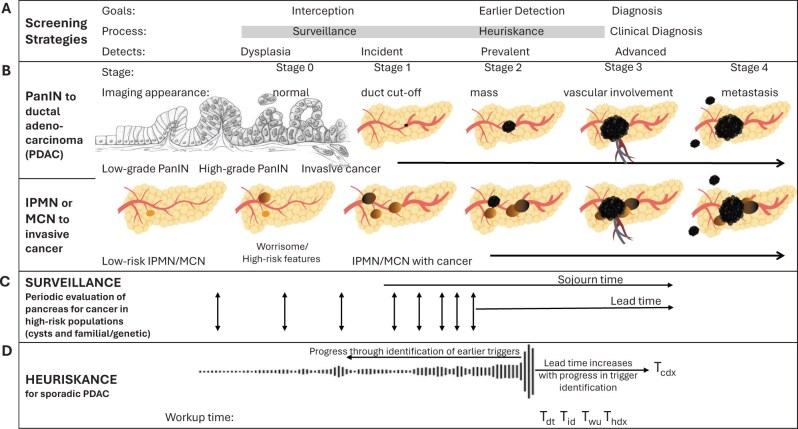
Strategies for the early detection of pancreatic cancer. **A**) The processes of surveillance and heuriskance are contrasted relative to their goals and the type of lesion targeted. Note vertical alignment with subsequent panels. **B**) Progression of 2 precursors to pancreatic cancer: pancreatic intraepithelial neoplasia (PanIN) to pancreatic ductal adenocarcinoma (PDAC) and intraductal papillary mucinous neoplasm (IPMN) or mucinous cystic neoplasm (MCN) to invasive cancer. The PanIN artwork was modified with permission from illustration copyright holder.^48^ Other rare forms of pancreatic cancer (eg, pancreatic neuroendocrine tumors) are not addressed in this commentary. **C**) The process of surveillance includes periodic imaging-based workups (**vertical arrows**) to detect the anatomical changes depicted in panel B. **D**) The process of heuriskance involves systematic screening for a signal (**vertical lines**) to identify individuals with a signal that meets the threshold for triggering a 1-time workup (**longer vertical line**). The benefit of the approach increases with increasing lead time between the trigger (T_dt_) and clinical diagnosis (T_cdx_) and depends on the nature of the trigger. Workup times from trigger detection (T_dt_) to identification (T_id_) to imaging-based workup (T_wu_) to heuriskance-based diagnosis (T_hdx_) are included in lead time and must be minimized.

Screening and ongoing surveillance of high-risk groups for cancer refers to the process of periodic testing of an asymptomatic population at approved intervals to detect early cancer. The United States Preventive Services Task Force has approved screening for cancers of breast, cervix, colon, lung, and prostate. Interception, or prevention of progression to invasive, fatal cancer, is the goal of screening (Figure 1, A). The earliest lesion that is the target of screening varies by organ: low-grade polyp (colon), high-grade dysplasia (cervix), invasive cancer (breast), and invasive cancer with high risk of progression (lung, prostate).

### Surveillance for pancreatic cancer 

Periodic, surveillance-based screening is currently conducted in 2 subgroups at high lifetime risk (3%-5% to 40%-60%) for pancreatic cancer (Figure 1, B and C)—that is, (1) individuals with a family history of pancreatic cancer or who carry a pancreatic cancer–predisposing gene (familial/genetic high-risk group) and (2) individuals with mucinous cysts (intraductal papillary mucinous neoplasms or mucinous cystic neoplasms) in the pancreas. Surveillance is possible in these 2 subgroups because the precursor lesions cause visible imaging abnormalities that change as disease progresses. Intraductal papillary mucinous neoplasms and mucinous cystic neoplasms have visible cystic lesions or dilated main pancreatic duct. Individuals with genetic or familial high risk often develop multifocal pancreatic intraepithelial neoplasms and intraductal papillary mucinous neoplasms^3^ that produce visible changes on magnetic resonance imaging and endoscopic ultrasound. Patients under surveillance in both groups undergo periodic evaluations of the pancreas by imaging until imaging features or cyst fluid analysis suggest high-grade dysplasia/early-stage cancer, at which time they undergo pancreatic resection.^4^

Remarkable progress has been made, with recent prospective studies in familial/high-risk groups showing that early detection and interception are possible,^5^^,^^6^ with improved survival.^4^ Surveillance-based screening in these 2 subgroups of patients faces numerous challenges,^4^^,^^7^^,^^8^ however, including optimizing surveillance, diagnostic evaluation and timing of surgery, and balancing overdiagnosis (unnecessary surgery for low-grade or benign pathologies) with failure to intercept cancer. The surveillance strategy in high-risk subgroups continues to evolve; an in-depth discussion, however, is beyond the scope of this commentary.

### Screening for sporadic pancreatic cancer

The surveillance approach described above is not applicable to 85% of pancreatic cancers because the rare pancreatic intraepithelial neoplasm that progresses to sporadic cancer is not visible on imaging. Earlier detection of even invasive resectable sporadic cancer faces major barriers, including low incidence, absence of approved biomarkers of early cancer, and the uncertain ability of imaging to identify prediagnostic lesions. Among the many barriers to screening for pancreatic cancer, the Achilles heel remains the disease’s low incidence; progress in biomarker development and imaging could potentially overcome the other barriers. Currently, the United States Preventive Services Task Force recommends against screening for sporadic pancreatic cancer,^9^ making it the only major cause of cancer mortality without an early-detection strategy.

### Heuriskance for earlier detection of pancreatic cancer: an emerging paradigm

The identification of novel markers of prevalent but asymptomatic cancer—for example, new-onset diabetes^10^ or artificial intelligence (AI)–generated clues from electronic health records (EHRs)^11-13^—point to possible ways forward for earlier detection of pancreatic cancer. We call this approach *heuriskance*, from the Greek word *heuriskein*, meaning to find or discover. Heuriskance refers to a population-based approach to finding cancer in persons with a heurisk, a systematically identified harbinger or early-warning sign of prevalent invasive cancer that patients and physicians may or may not be aware of and do not attribute to cancer. The heurisk would trigger a 1-time workup for prevalent cancer. Although the goal of both heuriskance and surveillance is to detect cancer at the earliest possible stage, the strategies differ in both approach and outcomes.

In contrast to surveillance, which is periodic, heuriskance involves a 1-time workup that is triggered by a discrete, nonrecurring event (Figure 1, D). Triggered workups have been part of clinical care in patients presenting with symptoms. Heuriskance differs from this “as-needed” approach by including a systematic search for triggers that meet criteria of a heurisk based on predictive thresholds, lead time, and scalability (discussed below). The subsequent workup of a trigger with currently available technology would include cross-sectional imaging and possibly endoscopic ultrasound to find and biopsy a suspicious lesion. The lesion detected would be prevalent, invasive cancer at an earlier stage than that diagnosed following onset of clinical symptoms. The episode of care, which may require short-term follow-up, and associated concern for cancer is for a limited time, after which patients without cancer would be reassured of the absence of cancer.

## Foundational elements of the heuriskance approach

### Tiered risk stratification: the Define-Enrich-Find approach

**Figure 2.**
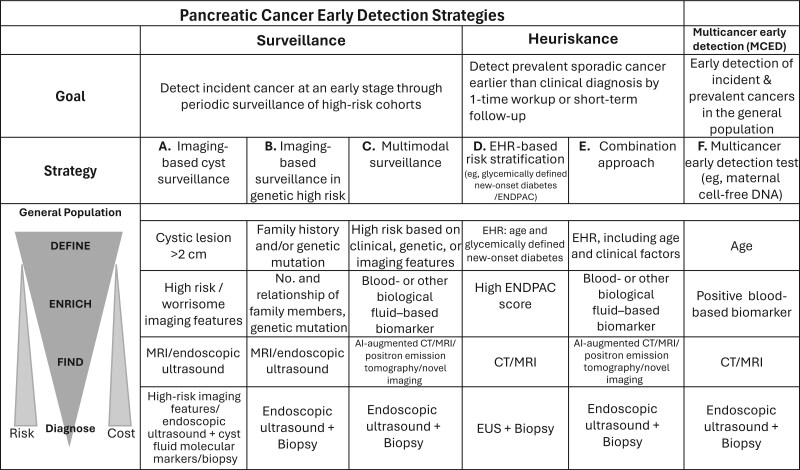
The Define-Enrich-Find approach to the early detection of pancreatic cancer. Specific strategies for surveillance and heuriskance approaches are compared by indicating their goal and the steps taken to define, enrich, and find an early-stage pancreatic cancer. **Figure inset**: The sequential approach systematically reduces the number of individuals who proceed to the next step by increasing the probability of having the disease, also referred to as increased risk, and increased positive predictive value of the process step. The sequential increase in risk allows increased cost for later vs earlier steps to achieve the cost-effectiveness of the strategy. **A, B**) Define-Enrich-Find surveillance strategies in use for high-risk cysts (**A**) and genetic high-risk populations (**B**). **C**) The addition of a blood-based or other biological fluid–based biomarker as an Enrichment step and artificial intelligence (AI)–based imaging to improve the Find step to surveillance strategies is in development and likely to be available soon. **D**) The heuriskance strategy of using electronic health records (EHRs) to define a high-risk population that is further enriched with additional EHR data and uses computed tomography or magnetic resonance imaging to find the lesion is being tested in the Early Detection Initiative trial. **E**) The addition of a biological fluid–based biomarker and/or AI-based imaging to the heuriskance strategy in panel D can improve the strategy. **F**) Multicancer early-detection tests that detect up to 50 cancers, including pancreatic cancer, are available and included for the sake of completeness. The strategy differs from surveillance because it targets an average-risk population and differs from heuriskance because it involves regular ongoing screening after a negative test.

To overcome the barrier of low cancer incidence, we proposed a Define-Enrich-Find approach^14^ wherein a **d**efined high-risk group is further **e**nriched to identify cohorts with higher-than-threshold risk in which imaging is used to **f**ind the lesion (Figure 2). The sequential approach allows for the application of complementary strategies to reduce the number of individuals who proceed to later steps by systematically selecting persons with increased risk to support endoscopic ultrasound, a procedure with higher risk and need for operators with advanced skill and training.

Define-Enrich-Find strategies are used in current surveillance-based approaches (Figure 2, A and B) and in surveillance and heuriskance approaches in development (Figure 2, C-E). Elements needed to “Define” a target population vary by strategy. The “Enrich” step is critical to the feasibility and cost-effectiveness of all strategies. Although in practice for other cancers it has generally been felt that blood—or other biological fluid—biomarker testing in the general population specifically for pancreatic cancer would not be feasible due to the high number of false positives. However, such biomarkers could be used in population subsets with higher-than-average risk to enrich cohorts to meet the threshold of risk for heuriskance. The “Find” step is essential for the success of a strategy because progression to a diagnostic workup depends on imaging visualization of small lesions.

The alternative strategy of early detection of many cancers, including pancreatic cancer, with a multicancer early detection test is included for completeness^5^ (Figure 2, F). It differs from surveillance in that it targets the general population and from heuriskance in that it requires regular screening. The problems and prospects of multicancer early-detection test use for early detection are under active investigation, but their discussion is beyond the scope of this commentary. They may have a role, however, in further enriching persons with a heurisk.

There are exciting advances in applying AI to standard images to “Find” early signals not visible to the human eye^15-17^ as well as novel approaches to molecular imaging^18^ that can be applied to early-detection strategies to markedly enhance their effectiveness. If time-cost optimized, imaging could also be used as the enriching step. Examples of this include use of abbreviated magnetic resonance imaging to screen for hepatocellular carcinoma in patients with cirrhosis^19^ or low-dose computed tomography (CT) for lung cancer screening in at-risk individuals who smoke.^20^

### The heurisk: defining features

A heurisk is a systemically identified early-warning sign or trigger for workup for cancer that meets all 3 criteria:

- Indicates a higher-than-threshold probability of having prevalent invasive pancreatic cancer
- Has a clinically meaningful lead time
- Identification is systematic and scalable

#### Indicates a higher-than-threshold probability of having prevalent invasive pancreatic cancer

We make an assumption that an early-warning sign of pancreatic cancer can be identified up to 3 years before the disease’s clinical diagnosis based on the example that elevated blood sugar can precede diagnosis within this time frame.^21^ In an average risk population, based on age and sex distribution^22^ and incidence in the United States,^2^ the 3-year incidence of pancreatic cancer in persons older than 50 years is 0.12% (0.04% per year). Cost-effectiveness studies define the threshold risk that would justify heuriskance as an approach to early detection. A study modeling glycemically defined new-onset diabetes–based heuriskance provides a framework for determining the minimal cost-effectiveness threshold.^23^ Based on reasonable assumptions, the authors concluded that a 3-year incidence of pancreatic cancer greater than 1% to 2% following the trigger would justify workup with CT and subsequent endoscopic ultrasound, if necessary.^23^ A heurisk with a 1.5% likelihood of prevalent cancer would result in a 10-fold to 15-fold higher risk than the general population. This threshold risk is comparable to risk in other cancer screenings (eg, the annual lung cancer risk for persons 50 years of age or older who have a 30-pack-year smoking history is 0.625%^20^; the incidence of breast cancer detected by digital mammography ranges from 0.14% to 0.76% in randomized trials^24^^,^^25^; colorectal cancer screening in individuals aged 50 years and older has a low detection rate of 0.146%^26^ for invasive cancer but the benefit of removing precancerous polyps justifies screening).

#### Has a clinically meaningful lead time

One of the challenges of screening for pancreatic cancer is that the cancer appears to be “silent” before diagnosis. Triggers such as symptoms or laboratory abnormalities that occur too close to the disease’s clinical diagnosis would not be clinically useful because they would lead to detection of advanced disease. To diagnose asymptomatic cancer, heuriskance needs a clinically meaningful lead time between the time of heurisk detection and clinical cancer diagnosis (Figure 1, D). Because the trigger is not immediately evident to patient or physician, the date it appears in the EHR (heurisk detection), the physician identifies the trigger, it is worked up, and the date of cancer diagnosis could be substantially delayed compared with heurisk detection; we base our discussions on the date of the trigger’s occurrence (heurisk detection). A priority for heuriskance is to minimize the delay between heurisk detection and cancer diagnosis. As technologies and research advance and earlier triggers are identified, the lead time for earlier diagnosis and stage of cancer identified through a heuriskance approach will improve (Figure 1, D).

**Figure 3.**
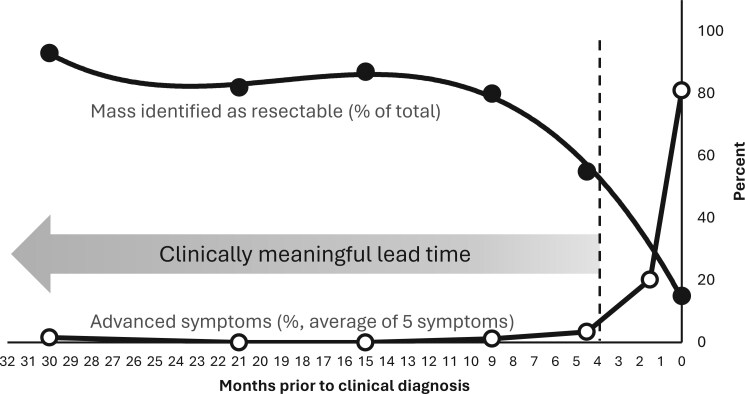
Clinically meaningful lead time for pancreatic cancer earlier detection. Computed tomography (CT) scans and symptom evaluation were available up to 3 years before a pancreatic adenocarcinoma diagnosis in a cohort of 128 patients.^31^  **Resectable mass**: CT scans taken for other purposes were retrospectively analyzed for resectability criteria and categorized in time intervals of 3-6, 6-12, 12-18, 18-24, and 24-35 months (Table S3^31^). At diagnosis, the authors reported 15% resectability. Results are plotted at 0 for at diagnosis and the midpoint of the time interval for prediagnostic values (closed circles). **Advanced symptoms**: The percentage of patients with documented abdominal pain, back pain, jaundice, fatigue, and anorexia were analyzed and categorized into time intervals of 0-3, 3-6, 6-12, 12-18, 18-24, and 24-36 months (Fig. S1^31^). At diagnosis, 19% of patients in a related cohort in which resection was possible were reported as asymptomatic (Table S3^31^). Results are plotted at 0 for at diagnosis, and values for the 5 symptoms were averaged and plotted at the time interval midpoint (open circles). A lead time of a minimum of 4 months was identified as being clinically meaningful, with an estimated 6% of patients with advanced symptoms compared with at least 81% at the time of diagnosis, and an estimated 50%-54% of patients with resectable disease compared with 15% at the time of diagnosis. Data from reference 31 reproduced with permission from the publisher.

It is well known that pancreatic cancer has an accelerated course in the few months before diagnosis. This behavior implies that detection of cancer before this acceleration begins would provide much greater benefit than lead time indicates. Retrospective studies show that most of this course acceleration occurs in the 4 months before diagnosis. Severe symptoms (abdominal pain, back pain, jaundice, anorexia, fatigue) begin in the 3 months before diagnosis^27^^,^^28^ (Figure 3). This period is also notable for clinically significant muscle wasting with consequent decrease in performance status.^29^^,^^30^ Retrospective review of CT scans in the months to years before diagnosis for unrelated indications show no stage IV disease and dramatically lower vascular involvement 3-6 months before diagnosis.^31^ Eighty percent of masses were identified as resectable 6-12 months before diagnosis, and 55% were identified at the 3-month to 6-month time point (Figure 3). All signals pointing to the presence of pancreatic cancer decrease with increasing lead time to diagnosis (eg, cancer antigen 19-9 sensitivity drops substantially in the 3-6 months before diagnosis, suggesting lower tumor burden^32^^,^^33^). Based on these observations, a clinically meaningful lead time could be defined at any time longer than 3-4 months. Patients with a clinically meaningful lead time of 4 months or more would be expected to be minimally symptomatic, would have normal performance status, and the proportion with stage III/IV disease would be notably lower than at current clinical diagnosis. In the recent large, prospective cohort study of individuals with glycemically defined new-onset diabetes, 70% had a lead time of at least 4 months.^10^

#### Identification is systematic and scalable

A heurisk is defined as being systematic and scalable rather than opportunistic and sporadic, typical of clinical workup in symptomatic patients. The trigger should either be widely available for testing in defined population subgroups (eg, a biomarker test in a high-risk patient) or identifiable through systematic periodic searches of EHRs (eg, glycemically defined new-onset diabetes).

#### Potential heurisks for earlier detection of pancreatic cancer

New-onset diabetes has been proposed as a trigger for evaluation for pancreatic cancer.^10^ Type 3C diabetes, which resembles type 2 diabetes but is caused by a developing cancer, is an often-unrecognized feature of pancreatic cancer.^34^ In a recent large, prospective cohort study, 0.62% of individuals with glycemically defined new-onset diabetes were diagnosed with pancreatic cancer within 3 years of diabetes onset.^10^ Ascertainment of glycemically defined new-onset diabetes was systematic among all members of a health-care system through periodic surveillance of EHRs for glycated hemoglobin levels.^10^ In another ongoing interventional trial (Early Detection Initiative for Pancreatic Cancer [ClinicalTrials.gov identifier NCT04662879]), glycemically defined new-onset diabetes with a high Enriching New-onset Diabetes for Pancreatic Cancer (ENDPAC) score was ascertained by systematic automated surveillance of EHRs.^35^ The ENDPAC score, which includes the rate of rise of glucose or glycated hemoglobin, change in weight, and age, has been shown to at least double the risk of pancreatic cancer in glycemically defined new-onset diabetes in retrospective studies.^21^^,^^36^ In this enriched cohort, a pilot study is testing the utility of CT scans to identify asymptomatic cancer.^35^ In other approaches, AI and natural language processing of EHR data identified individuals at high risk of prevalent cancer.^37^ These opportunities highlighted the need for guidelines for the development of this unprecedented approach to cancer screening.

### Success metrics

#### At-screen detection

Unlike the surveillance-based approach, where the goal is interception at high-grade dysplasia/stage I cancer, heuriskance aims to find cancer earlier than clinical diagnosis (Figure 1, A). Heuriskance aims to breach the seemingly impenetrable gap between inception of cancer and its clinical diagnosis; its goals are, at first, to improve survival (as in clinically diagnosed cancer) and eventually to improve cancer mortality (as with screening) by progressing the goal for detection from premetastatic to curable disease, with technological improvements over time (Figure 1, D). To appreciate the promise of heuriskance, novel metrics are needed to measure its success:

- **Proportion asymptomatic/minimally symptomatic patients.** To be considered a screening modality, heuriskance must identify patients with “no noticeable symptoms of cancer.” Currently, approximately 90% of patients with pancreatic cancer are symptomatic at diagnosis. Retrospective studies suggest that if heuriskance for pancreatic cancer is initiated at a minimum clinically meaningful lead time of 4 months or more, 85%-95% of patients would be asymptomatic/minimally symptomatic^28^^,^^30^^,^^31^ (Figure 3).
- **Proportion of stage III/IV cancers.** Currently, 85% of patients have stage III/IV disease at diagnosis. Many successful screening programs in other organs have achieved at least 20% later-stage disease reduction (eg, low-dose CT for lung cancer).^38^ A reasonable metric is less than 68% of patients at the time of the trigger have stage III/IV disease (0.8 × 85% = 68%).

#### Outcome metrics

Although the initial goals of heuriskance are detection of asymptomatic/minimally symptomatic cancer and stage shifting, these goals must translate to improved survival and mortality to establish clinical utility. Three- or 5-year survival in screen-detected cancer can be affected by lead time or overdiagnosis bias. The concern for diagnosing indolent, nonprogressive invasive cancer (overdiagnosis bias) is generally thought of as unlikely for sporadic pancreatic cancer. Lead time bias, however, is a real threat to misinterpreting survival data because there is evidence that a heurisk such as glycemically defined new-onset diabetes precedes clinical diagnosis of pancreatic cancer by 2 to 3 years. Several metrics represent intermediate milestones:

- **Proportion of stage III/IV cancers.** This metric can be compared between 2 arms for a randomized heuriskance trial or compared with historical data in a single-arm study.^35^^,^^39^^,^^40^ Overdiagnosis is a potential concern with this metric in a single-arm study. Comparing the heuriskance and control arms in a randomized trial for the total cancer incidence rate would be required to confirm lack of overdiagnosis.^39^
- **Cumulative incidence of stage III/IV cancers.** This metric can be compared between 2 arms in a randomized trial.^41^ There is no overdiagnosis bias in evaluating stage shift, but it requires a larger sample size than does a study using the proportion of stage III/IV cancers as the endpoint. It is a necessary but not sufficient metric for mortality reduction.
- **Lead time–adjusted 1-, 3-, and 5-year survival.** Survival metrics can be adjusted to account for lead time bias when the lead time distribution is known from observational studies, such as that used to query glycemically defined new-onset diabetes.^10^ This lead time can be subtracted from the observed survival time in an interventional screening study. In a randomized controlled trial or a single-arm study compared with a compatible observational study, the difference between the time of heurisk detection to death with and without the screening intervention is the survival benefit without lead time bias because survival time starts from trigger date heurisk detection, not from date of diagnosis. It should be noted that overdiagnosis bias remains with this metric; however, overdiagnosis is judged not to be a major concern for pancreatic cancer.
- **Cumulative mortality incidence rate.** Cumulative cancer mortality incidence as a primary outcome of a randomized trial is the gold standard for evaluating clinical utility of a screening strategy but requires a large sample size and is not feasible for low-incidence diseases. A randomized controlled trial in a pancreatic cancer average-risk population with an effect size of 20% mortality reduction, 90% power, and 5-year follow-up requires a sample size of approximately 570 000 for a cumulative mortality endpoint (methodology as per^42^). This metric was judged as infeasible, even for a multicancer early-detection randomized clinical trial.^41^ For a heurisk-enriched population with a 1.5% prevalence, a trial requires approximately 79 000 persons randomly assigned. A more feasible approach to an EHR-based heuriskance trial is a pragmatic trial in multiple large health-care systems with site-based- or health-care-based randomization of heuriskance vs standard-of-care approaches.

## A phased approach to heuriskance

We applied the adopted 5-phase guidelines^43^ for developing heuriskance strategies compared with a surveillance strategy (Table 1). These strategies differ in phases 1 through 3 when a biomarker is included as the trigger (multimodal and combination approaches) compared with EHR approaches (EHR-only)

**Table 1.** A phased approach to the development of pancreatic cancer surveillance and heuriskance strategies.^a^

Phases for biomarker development^43^	Surveillance	Heuriskance	
	Multimodal surveillance (Figure 2, C)	EHR-based risk stratification (Figure 2, D)	Combination approach (Figure 2, E)
Starting population	High-risk subpopulation: Family history or predisposition gene carrier Cyst with worrisome/high-risk features	All participants >50 y of age	All participants >50 y of age
Indication for CT imaging workup	Positive signal for blood (or other biological fluid) biomarkers	Best practice alert from EHR indicates clinical high-risk group (eg, no evidence of disease, high ENDPAC score, other EHR-based predictor)	Best practice alert from EHR indicates blood (or other biological fluid)-based test; positive signal for biomarker indicates CT imaging
Phase 1. Preclinical exploratory: Provides promising leads with technical feasibility and analytical validity	Objective: Develop an assay for candidate blood (or other biological fluid) biomarkers associated with pancreatic cancer. Criteria for proceeding: Assay meets analytical standards (reproducibility, detection limit, transportability) Better than or complementary to cancer antigen 19-9	Objectives: Develop an algorithm to define a pancreatic cancer high-risk population. Precisely define EHR predictors. Customize the algorithm for EHR configuration. Criteria for proceeding: Algorithm demonstrates concordance with manual verification of output. Percentage of population with algorithm-defined predictors meets cost-effectiveness parameters.	Objective: Develop an assay for candidate biomarker associated with pancreatic cancer. Criteria for proceeding: Assay meets analytical standards (reproducibility, detection limit, transportability) Better than or complementary to cancer antigen 19-9
Phase 2. Clinical assay and clinical validation: Provides initial clinical validation with clinical-grade assay	Objective: Demonstrate that candidate biomarkers are associated with high-grade dysplasia/stage I pancreatic cancer using clinical-grade or high-quality research assay. Characteristics of study: Retrospective case control Criterion for proceeding: Better than or complementary to cancer antigen 19-9	Objective: Demonstrate that the algorithm identifies a pancreatic cancer high-risk group. Characteristics of study: Retrospective EHR-based cohort Criteria for proceeding: Estimated positive predictive value meets cost-effectiveness–identified parameters. Clinically meaningful lead time meets specifications.	Objective: Demonstrate that candidate biomarkers are associated with pancreatic cancer using a clinical-grade or high-quality research assay. Characteristics of study: Retrospective case control Criterion for proceeding: Better than or complementary to cancer antigen 19-9
Phase 3. Retrospective longitudinal: Provides retrospective clinical validation on asymptomatic population a key step before clinical utility evaluation	Objective: Validate the assay and define test positive rule. Characteristics of study: Prospectively collected specimens from target population, retrospective blinded evaluation^44^ Criteria for proceeding: TPR and FPR satisfy the benefit-to-harm condition: *p* × TPR × B_+D_ − *p* × (1 – TPR) × C_-D_ + (1 − *p*) × (1 – FPR) × B_-N_ – (1−*p*) × FPR × C_+N_ −C_b_ > 0, where *p* is the incidence rate in the population, TPR = true positive rate, FPR = false positive rate, B = benefit, and C = cost/harm^45^ The benefit-to-harm condition provides a framework but requires cost-effectiveness analysis to determine benefits and costs/harms.	Objective: Validate algorithm, decision rule, risk, and lead time distribution. Characteristics of study: Prospective EHR-based cohort Criteria for proceeding: Positive predictive value meets parameters for population enrichment. Lead time meets parameters for being clinically meaningful. True positive rate and false positive rate satisfy the benefit-to-harm condition (no assay cost or harm to false negatives): *p* × TPR × B_+D_ – (1 – *p*) × FPR × C_+N_ > 0, where *p* is the incidence rate in the population, TPR = true positive rate, FPR = false positive rate, B = benefit, and C = cost/harm^45^ (Higher true positive rate is the goal for improvement but not a metric for success.)	Objective: Validate assay and fix test positive rule. Characteristics of study: Prospectively collected specimens from target high-risk population, retrospective blinded evaluation Criteria for proceeding: Lead time meets parameters for being clinically meaningful. TPR and FPR satisfy the benefit to harm condition: *p* × TPR × B_+D_ − *p* × (1 – TPR) × C_-D_ + (1−*p*) × (1 – FPR) × B_-N_ – (1 – *p*) × FPR × C_+N_ − C_b_ > 0, where *p* is the incidence rate in the population, TPR = true positive rate, FPR = false positive rate, B = benefit, and C = cost/harm^45^
Phase 4. Prospective screening: Prospective clinical validation	Objective: Confirm positive predictive value, stage shift, and surgery rate. Characteristics of study: Prospective randomized trial with control arm at multiple sites preferred; single-arm study possible Criterion for proceeding: Benefit-to-harm condition >0 determined by detection rate and positive predictive value (function of screening program true positive rate, false positive rate, incidence rate, and surgery and resection rates)	Objective: Confirm positive predictive value, stage shift, and lead time–adjusted survival benefits. Characteristics of study: Prospective randomized trial with control arm at multiple sites Criteria for proceeding: Positive predictive value meets parameters for population enrichment. Stage shift meets parameters for reduction in proportion or incidence of stage III/IV cancers at screen detection among positive tests. Detection rate (nonessential) is the proportion of cancers detected. Lead time–adjusted survival (1-y, 3-y, 5-y) meets parameters for increase over controls/historic controls among persons who test positive. (Note: Criteria 1, 2, and 4 measured on patients who test positive as stopping rule. Criteria 2-4 when measured on all patients [ie, individuals who test positive or negative], is the goal for improvement but not a metric for success.)	Objective: Confirm positive predictive value, stage shift, and lead time–adjusted survival benefits. Characteristics of study: Prospective randomized trial with control arm at multiple sites preferred; single-arm study possible Criteria for proceeding: benefit-to-harm condition >0 determined by detection rate and positive predictive value (function of screening program true positive rate, false positive rate, and incidence rate); stage shift meets parameters for reduction in the proportion or incidence of stage III/IV cancers at screen detection; and lead time–adjusted survival (1-y, 3-y, 5-y) meets parameters for increase over controls/historic controls.
Phase 5. Cancer control: Tests clinical utility	Objective: Demonstrate reduction in pancreatic cancer mortality and overall mortality. Characteristics of study: Randomized clinical trial Criterion for success: Meets predetermined criteria for percentage reduction in pancreatic cancer–specific mortality and overall mortality	Objective: Demonstrate reduction in pancreatic cancer mortality. Characteristics of study: Randomized pragmatic trial with multiple health-care systems Criterion for success: Meets predetermined criteria for percentage reduction in pancreatic cancer–specific mortality	Objective: Demonstrate reduction in pancreatic cancer mortality Characteristics of study: Randomized clinical trial (may not be feasible for average-risk population; may rely on randomized pragmatic trial) Criterion for success: Meets predetermined criteria for percentage reduction in pancreatic cancer–specific mortality

Abbreviations: CT = computed tomography; EHR = electronic health record; ENDPAC = Enriching New-onset Diabetes for Pancreatic Cancer.

a The phases for biomarker development^43^ are applied to the multimodal surveillance Define-Enrich-Find strategy and the heuriskance EHR-based and combination Define-Enrich-Find approaches. The multimodal surveillance and heuriskance combination approaches include a blood-based or other biological fluid–based biomarker, whereas the EHR-based heuriskance approach relies on EHRs for the Define and Enrich steps. A major difference between the heuriskance EHR-based approach and the other strategies involving fluid-based biomarkers is the benefit-to-harm condition in phase 3 can be achieved without considering assay cost or harm of false negatives in the EHR-based approach.

### Phases 1–3 of a biomarker in a surveillance, high-risk group strategy

In phase 1, a promising biomarker is identified. In phase 2 the biomarker should perform as well as or better than cancer antigen 19-9 in a retrospective case control study. In phase 3, the PRoBE design^44^ is used to test the performance characteristics of the biomarker against specimens prospectively collected from the target population.

### Phases 1–3 in the development of a heuriskance strategy with EHR-only approaches

For EHR-only approaches, the ability to identify the trigger is confirmed in a phase 1 study, and in phase 2, the algorithm is refined to achieve the necessary risk threshold. Retrospective studies should provide a reasonable estimate of expected lead time distribution. In phase 3, the electronic algorithm is tested prospectively in different health-care systems, and risk and lead time distribution are confirmed.

### Development of phases 4 and 5 of a heuriskance strategy

To evaluate the potential clinical utility and decide whether a phase 4 study is warranted, a benefit-to-harm condition analysis should be conducted for any surveillance or heuriskance-based screening test.^45^ Benefit-to-harm condition analysis considers the true and false positive rates, the benefit of true positives and negatives, the cost or harm of false positives and negatives, and the cost of identifying and measuring the trigger. A heuriskance blood-based or imaging-based approach will have values for all the terms in the benefit-to-harm condition analysis because the participants will be aware of the test results and triggers will have real costs for the assay and imaging. For an EHR-based trigger, the harm of false negatives and benefit of true negatives are minimal because the standard of care is not altered for participants with negative triggers. The routine run of software to identify individuals with a positive trigger is essentially cost-free after initial setup costs. Therefore, in the EHR-only approach, the benefit-to-harm condition is reduced to becoming equivalent to the questions, “How many workups to detect one cancer would be cost-effective?” and “What positive predictive value will justify the use of the test?” This unique feature of heuriskance EHR-based triggers makes it easier to satisfy the benefit-to-harm condition and meet milestones.

For phase 4, a prospective randomized trial to confirm the lead time distribution, stage shift, and lead time–adjusted survival benefit is preferred and more easily achievable with an EHR-based approach than with either a surveillance or biomarker approach, where a single-arm study may be necessary. The benefit-to-harm condition analysis should be updated with phase 4 study data to ensure that the benefit-to-harm condition is greater than 0 before launching a phase 5 trial. Phase 5 is a randomized controlled trial to establish mortality benefit. A randomized pragmatic trial is likely to be required for low-incidence diseases.

### Milestones for a heuriskance-based approach

As prospective heuriskance studies are undertaken (phases 4-5), we propose moving through the metrics at screen detection (proportion of asymptomatic patients, proportion of patients with stage III/IV disease or cumulative stage III/IV disease incidence) to the outcome metrics (lead time–adjusted 1-year, then 3-year, then 5-year adjusted survival, mortality incidence reduction) progressively to assess any heuriskance-based strategy. If these goals are not met, they can serve as early stopping metrics for prospective trials.

## Limitations

The heuriskance-based strategy faces a unique set of challenges. The success of the strategy in the research and clinical setting not only will depend on the lead time distribution of the trigger but also includes the time that elapses from the date of trigger occurrence to diagnosis. All lead time advantage can be lost by delays in diagnosis. In the research setting, there can be clinically significant delays in consenting patients and scheduling research procedures, steps that are not relevant in the clinical setting.

We describe the approach as involving a 1-time workup. It may, in some cases, require a second imaging study in 4-6 months to rule out new or progressive abnormalities missed at first workup. The risk of cancer rapidly decreases with time, however, and returns to a baseline, incorporating the risk of having diabetes^46^^,^^47^ and individual factors, such as age and genetics. To maximize the benefit of a heuriskance-based strategy, imaging modalities should have a high sensitivity for early cancer because it assumes that imaging can identify pancreatic cancer up to 3 years before clinical diagnosis. The poor performance of imaging beyond 3-6 months of lead time is well known, and it is unknown whether endoscopic ultrasound can identify prediagnostic disease. To fully realize the potential of this strategy for early detection, substantial and simultaneous advances in biomarker development and imaging of prediagnostic cancer are necessary. Major work is being done on both fronts, providing hope and optimism for overcoming this limitation.

Whether earlier detection is sufficient to improve cancer mortality in pancreatic cancer is an open question. Like chemotherapies for metastatic cancer, incremental advances improve survival and lay the groundwork for more substantial impact on mortality. We strongly believe that if there were substantial improvements in lead time–adjusted 1-year and especially 3-year survival, trials of recurrence prevention strategies could help achieve the goal of mortality benefit for pancreatic cancer.

## Conclusion

In summary, we propose a novel paradigm for a systematic approach to earlier detection of pancreatic cancer, arguably the toughest problem in cancer early detection. Previously thought to be silent, the presymptomatic phase produces metabolic and soft tissue changes that provide a window of opportunity in which to screen for prevalent but asymptomatic disease. A well-chosen heurisk could be incorporated into EHRs to generate automated “early-warning sign” alerts that identify a large proportion of cancers with a clinically meaningful lead time. Glycemically defined new-onset diabetes is a documented heurisk, and other patterns have been described that are recognizable by AI. Developments in the biomarker and AI-assisted imaging fields will further enhance the feasibility of the approach, and improvements in treatment will synergize to expand the impact on mortality. Heuriskance has the advantage of limiting the period of concern to a single episode of care or short-term follow-up at most.

Currently, heuriskance is the most feasible and fastest way forward for early detection of pancreatic cancer. Heuriskance aims to minimize the proportion of patients with advanced disease and maximize treatment options, with subsequent improvements in survival and the hope that incremental advances will eventually affect mortality. We foresee improvements in mortality occurring through progressively increasing lead time to diagnosis and concurrent improvements in upfront therapy and prevention of recurrence. These approaches challenge our current paradigms for early detection. As we envision the possibility of systematic implementation of a heuriskance approach, we provide guidance for development and milestones to measure incremental progress in this field. The proposed heuriskance paradigm could be adopted to earlier detection for other cancers where direct screening of average-risk populations is challenging.

## Data Availability

No new data were generated for this commentary. Datasets were derived from sources in the public domain (https://seer.cancer.gov/statistics-network/explorer/).
